# Chlorido­[(1,2,5,6-η)-cyclo­octa-1,5-diene](1-ethyl-4-isobutyl-1,2,4-triazol-5-yl­idene)rhodium(I)

**DOI:** 10.1107/S2414314624007041

**Published:** 2024-07-23

**Authors:** Timothy G. Lerch, Michael Gau, Daniel R. Albert, Edward Rajaseelan

**Affiliations:** a Department of Chemistry Millersville University,Millersville PA 17551 USA; b Department of Chemistry, University of Pennsylvania, Philadelphia, PA 19104, USA; Goethe-Universität Frankfurt, Germany

**Keywords:** crystal structure, rhodium, N-heterocyclic carbenes, neutral transition-metal complexes

## Abstract

A new neutral triazole-based N-heterocyclic carbene rhodium(I) complex [RhCl(C_8_H_12_)(C_8_H_15_N_3_)], has been synthesized and structurally characterized. The complex crystallizes with two mol­ecules in the asymmetric unit. The central rhodium(I) atom has a distorted square-planar coordination environment, formed by a cyclo­octa-1,5-diene (COD) ligand, an N-heterocyclic carbene (NHC) ligand, and a chlorido ligand. The bond lengths are unexceptional. A weak inter­molecular non-standard hydrogen-bonding inter­action exists between the chlorido and NHC ligands.

## Abstract 2

A new neutral triazole-based N-heterocyclic carbene rhodium(I) complex [RhCl(C_8_H_12_)(C_8_H_15_N_3_)], has been synthesized and structurally characterized. The complex crystallizes with two mol­ecules in the asymmetric unit. The rhodium center has a distorted square-planar conformation, formed by a cyclo­octa-1,5-diene (COD) ligand, an N-heterocyclic carbene (NHC) ligand, and a chloride ligand.

## Graphical abstract

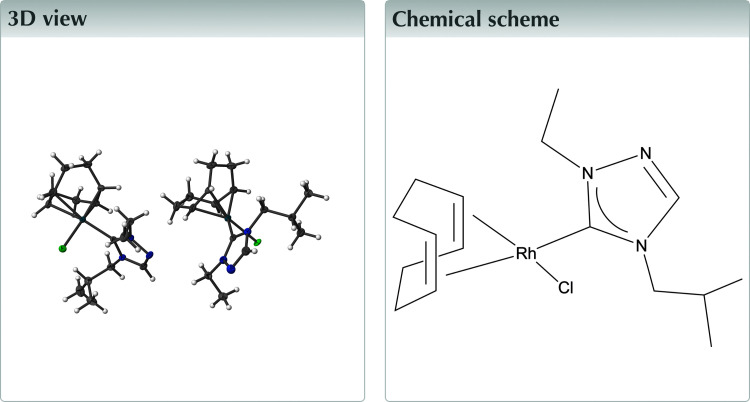

## Structure description

Numerous and ever-increasing applications of N-heterocyclic carbenes (NHCs) as supporting ligands in late transition-metal catalysis have been reported (Diez-González *et al.*, 2009 ▸; Cazin, 2013 ▸; Rovis & Nolan, 2013 ▸; Ruff *et al.*, 2016 ▸; Zuo *et al.*, 2014 ▸). Their catalytic activity in the transfer hydrogenation of ketones and imines has also been studied and reported (Albrecht *et al.*, 2002 ▸; Gnanamgari *et al.*, 2007 ▸). The NHC ligands can be tuned sterically and electronically by having different substituents on the nitro­gen atoms (Diez-González & Nolan, 2007 ▸; Gusev, 2009 ▸). Though many imidazole- and tri­azole-based NHC rhodium and iridium complexes have been synthesized and structurally characterized (Herrmann *et al.*, 2006 ▸; Wang & Lin, 1998 ▸; Chianese *et al.*, 2004 ▸; Nichol *et al.*, 2009 ▸, 2010 ▸, 2011 ▸, 2012 ▸; Idrees *et al.*, 2017*a* ▸,*b* ▸; Rood *et al.*, 2021 ▸; Rushlow *et al.*, 2021 ▸; Newman *et al.*, 2021 ▸; Castaldi *et al.*, 2021 ▸; Maynard *et al.*, 2023 ▸; Lerch *et al.*, 2024 ▸), new complexes with different substituents (‘wing tips’) on NHC ligands are being synthesized to study their effects in the catalytic properties of these complexes.

**Figure 1:**
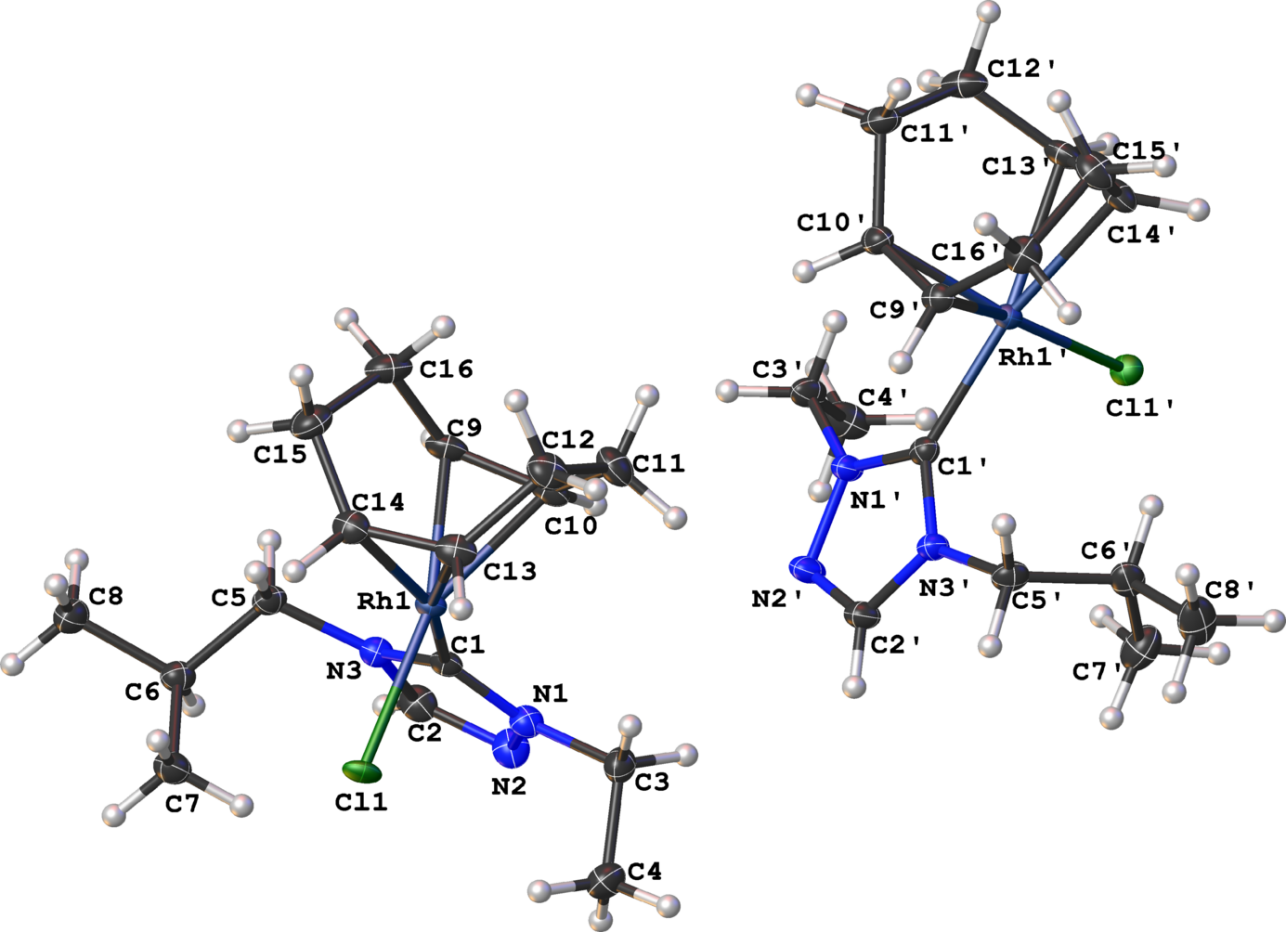
Asymmetric unit of the title compound (**3**) showing the two mol­ecular units. Displacement ellipsoids are drawn at the 50% probability level.

**Figure 2:**
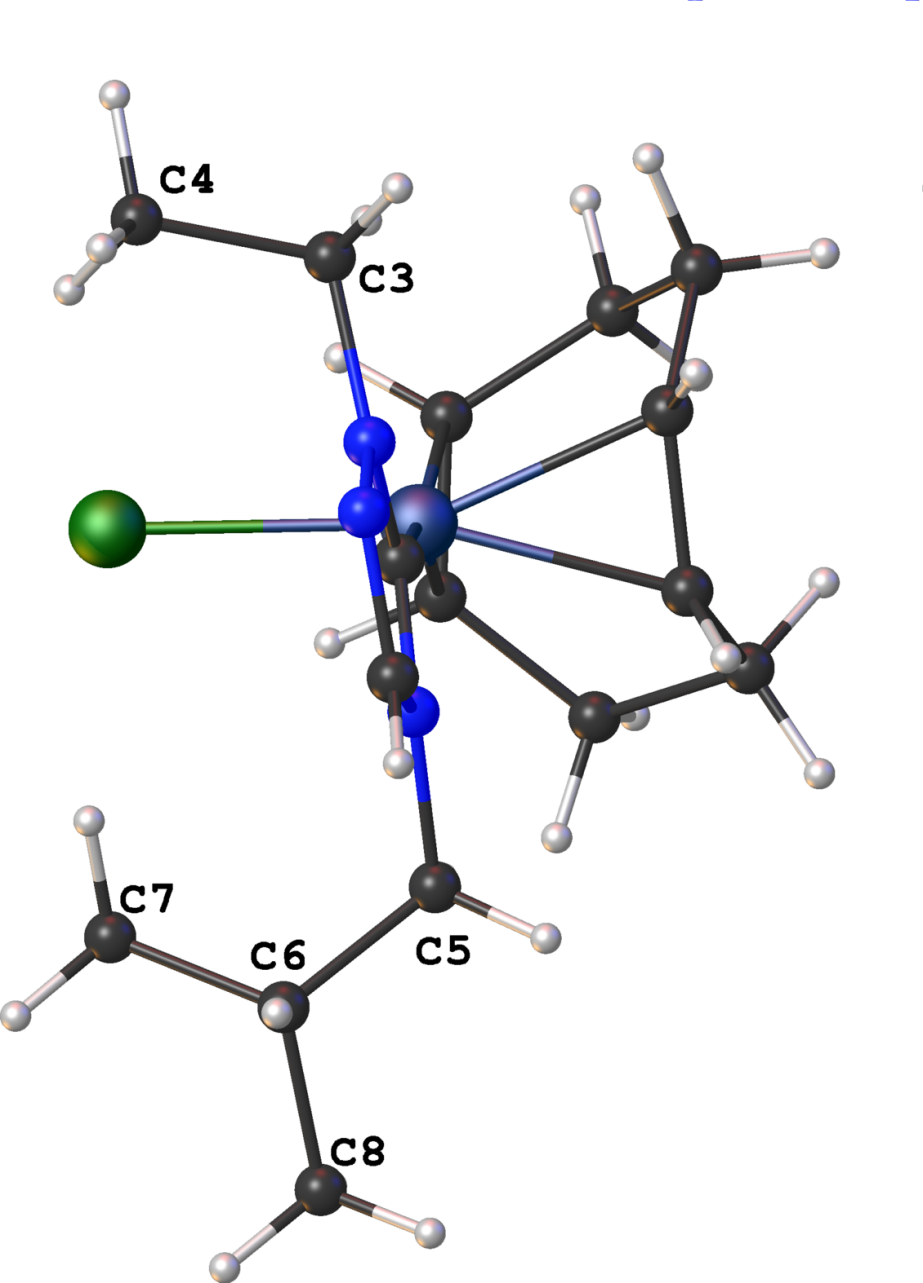
View of one mol­ecule of the title compound (**3**) showing the ethyl and isobutyl wingtips oriented on the same side of the NHC ring and away from the COD ligand.

**Figure 3:**
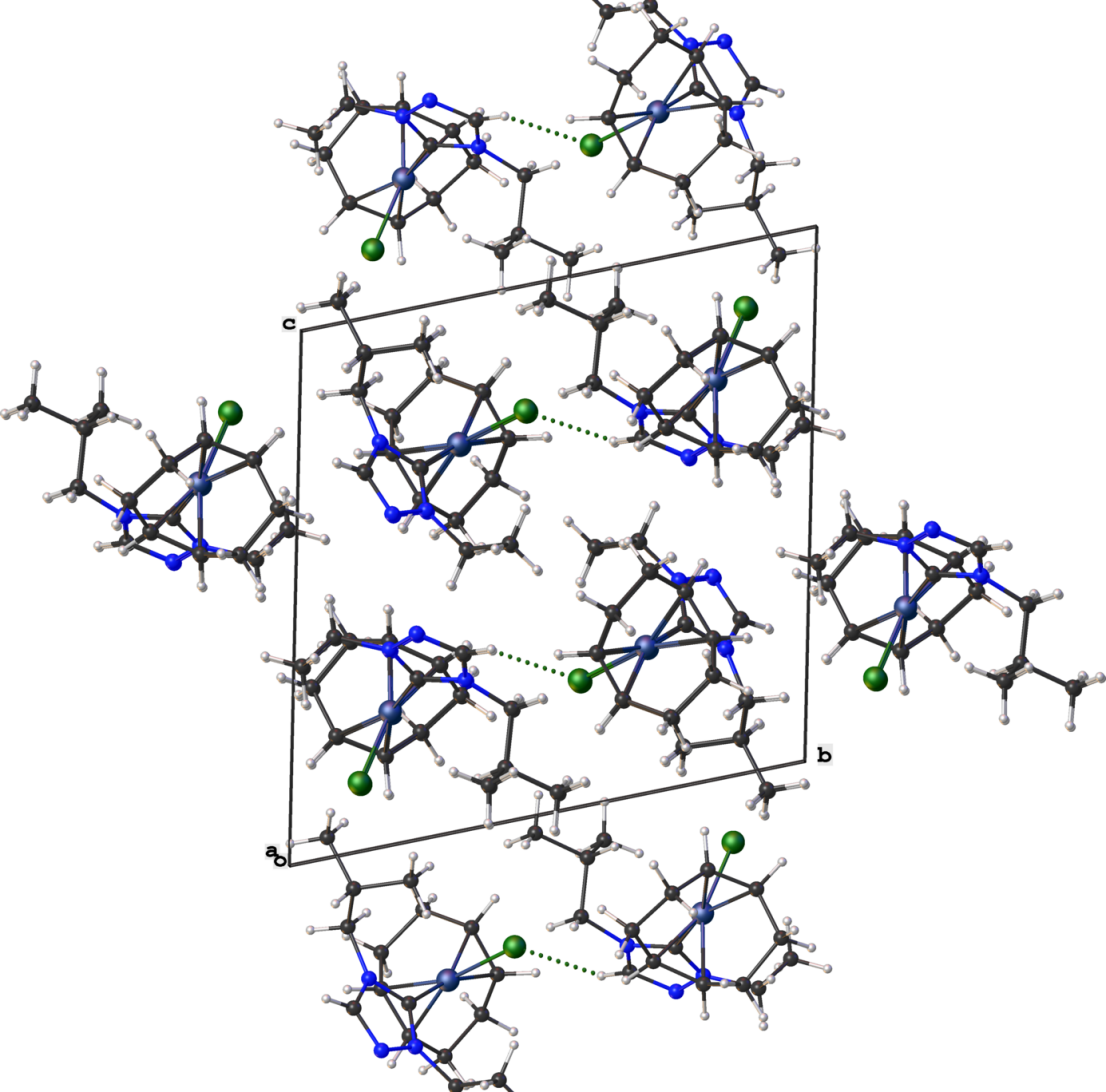
Crystal packing diagram of the title compound (**3**) viewed along the *a* axis. C—H⋯Cl non-standard hydrogen-bonding inter­actions are shown as dotted green lines.

The compound [RhCl(C_8_H_12_)(C_8_H_15_N_3_)] (**3**), as illustrated in Fig. 1 ▸, crystallizes in the triclinic space group *P*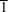 with two mol­ecules in the asymmetric unit. No solvent mol­ecules were found in the structure. The coordination sphere around the Rh^I^ ion is formed by the bidentate COD, NHC, and chlorido ligands, resulting in a distorted square-planar shape. The carbene atom, C1, deviates from the expected *sp*^2^ hybridization in that the N1—C1—N3 bond angle in the triazole-based carbene is 102.77 (17)° [N1′—C1′—N3′ is 102.45 (16)°]. Other selected bond lengths and angles in the structure are: Rh1—C1(NHC) = 2.020 (2) Å, Rh1′—C1′(NHC) = 2.012 (2) Å, Rh1—Cl1 = 2.3846 (5) Å, Rh1′—Cl1′ = 2.3887 (5) Å, C1—Rh1—Cl1 is 88.36 (5)°, and C1′—Rh1′—Cl1′is 88.57 (6)°. The two substit­uent ‘wing tips’ in the NHC (N1-ethyl and N3-isobut­yl) are oriented in a *syn* arrangement with respect to one-another. The ethyl and isobutyl ‘wingtips’ are both oriented away from the COD ring as illustrated in Fig. 2 ▸. The packing, as illus­trated in Fig. 3 ▸, is consolidated through weak non-standard hydrogen-bonding inter­action between the NHC and chlorido ligands of adjacent mol­ecules. The non-standard hydrogen-bonding inter­actions are summarized in Table 1 ▸ and shown as dotted green lines in Fig. 3 ▸.

## Synthesis and crystallization

**Figure 4:**
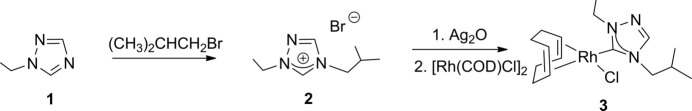
Reaction scheme for the synthesis of the title compound (**3**).

1-Ethyl-1,2,4-triazole (**1**) was purchased from Matrix Scientific. All other compounds used in the syntheses, detailed in Fig. 4 ▸, were obtained from Sigma-Aldrich and Strem and used as received; all syntheses were performed under a nitro­gen atmosphere. NMR spectra were recorded at room temperature in CDCl_3_ on a 400 MHz (operating at 100 MHz for ^13^C and 162 MHz for ^31^P) Varian spectrometer and referenced to the residual solvent peak (δ in p.p.m.). The title compound (**3**) was crystallized by slow diffusion of pentane into a CH_2_Cl­_2_ solution.

**1-Ethyl-4-isobutyl-1,2,4-triazolium bromide (2):** 1-Ethyl-1,2,4-triazole (**1**) (1.020 g, 10.50 mmol) and excess 1-bromo-2-methyl­propane (5.436 g, 39.67 mmol) were added to toluene (15 ml), and the mixture was refluxed in the dark for 48 h. After the mixture was cooled, the white solid was filtered, washed with ether, and dried under vacuum. Yield: 0.625 g (25.4%). ^1^H NMR: δ 11.71 (*s*, 1 H, N—C5H—N), 8.62 (*s*, 1 H, N—C3H—N), 4.90 (*q*, 2 H, N—CH_2_ of eth­yl), 4.38 (*d*, 2 H, N—CH­_2_ of isobut­yl), 2.32 (*m*, 1 H, CH of isobut­yl), 1.64 (*t*, 3H, CH_3_ of eth­yl), 1.03 (*d*, 6 H, CH_3_ of isobut­yl). ^13^C NMR: δ 143.49 (N—C5—N), 142.66 (N—C3—N), 55.46 (N—CH_2_ of isobut­yl), 48.50 (N—CH_2_ of eth­yl), 29.31 (CH of isobut­yl), 19.49 (CH­_3_ of isobut­yl), 14.18 (CH_3_ of eth­yl).

**Chlorido­[(1,2,5,6-η)-cyclo­octa-1,5-diene](1-ethyl-4-iso­butyl-1,2,4-triazol-5-yl­idene)rhodium(I) (3):** Triazolium bromide (**2**) (0.095 g, 0.406 mmol) and Ag_2_O (0.047 g, 0.203 mmol) were stirred at room temperature in the dark for 1 h in CH_2_Cl_2_ (10 ml). The mixture was then filtered through Celite into [Rh(cod)Cl]_2_ (0.100 g, 0.203 mmol), and stirred again in the dark for 1.5 h. The resulting solution was filtered through Celite and the solvent was removed under reduced pressure in a rotary evaporator. The yellow solid product (**3**) was dried under vacuum. Yield: 0.149 g (92%). ^1^H NMR: δ 7.82 (*s*, 1 H, N—C3H—N), 4.74 (*q*, 2 H, N—CH_2_ of eth­yl), 4.66 (*d*, 2 H, N—CH_2_ of isobut­yl), 4.30 (*m*, 2 H, CH of COD), 4.20 (*m*, 2H, CH of COD), 3.37, 3.24 (*m*, 4 H, CH_2_ of COD), 2.60, 2.46 (*m*, 4 H, CH_2_ of COD), 2.32 (*m*, 1 H, CH of isobut­yl), 1.59 (*t*, 3 H, CH_3_ of eth­yl), 1.08 (*d*, 6 H, CH_3_ of isobut­yl). ^13^C NMR: δ 184.95 (*d*, Rh—C, *J*_C—Rh_ = 50.9 Hz), 142.29 (N—C3H—N), 99.43,99.36, 99.13, 99.06 (CH of COD), 56.21 (N—CH_2_ of isobut­yl), 48.01 (N—CH_2_ of eth­yl), 47.91, 33.29,32.45,30.80,29.30 (CH_2_ of COD), 29.13 (CH of isobut­yl), 20.02 (CH­_3_ of isobut­yl), 15.36 (CH_3_ of eth­yl).

## Refinement

Crystal data, data collection and structure refinement details are summarized in Table 2 ▸.

## Supplementary Material

Crystal structure: contains datablock(s) I. DOI: 10.1107/S2414314624007041/bt4153sup1.cif

Structure factors: contains datablock(s) I. DOI: 10.1107/S2414314624007041/bt4153Isup2.hkl

CCDC reference: 2371669

Additional supporting information:  crystallographic information; 3D view; checkCIF report

## Figures and Tables

**Table 1:** Hydrogen-bond geometry (Å, °)

*D*—H⋯*A*	*D*—H	H⋯*A*	*D*⋯*A*	*D*—H⋯*A*
C2′—H2′⋯Cl1^i^	0.95	2.62	3.502 (2)	155

Symmetry code: (i) 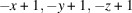.

**Table 2:** Experimental details

Crystal data	
Chemical formula	[RhCl(C_8_H_12_)(C_8_H_15_N_3_)]
*M*_r_	399.76
Crystal system, space group	Triclinic, *P* 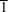
Temperature (K)	100
*a*, *b*, *c* (Å)	9.6253 (1), 13.6771 (2), 13.7938 (2)
α, β, γ (°)	76.410 (1), 83.455 (1), 80.345 (1)
*V* (Å^3^)	1734.78 (4)
*Z*	4
Radiation type	Mo *K*α
μ (mm^−1^)	1.14
Crystal size (mm)	0.30 × 0.23 × 0.15

Data collection	
Diffractometer	Rigaku XtaLAB Synergy-S
Absorption correction	Multi-scan (*CrysAlis PRO*; Rigaku OD, 2024 ▸)
*T*_min_, *T*_max_	0.770, 1.000
No. of measured, independent and observed [*I* > 2σ(*I*)] reflections	52942, 8619, 7885
*R*_int_	0.037
(sin θ/λ)_max_ (Å^−1^)	0.667

Refinement	
*R*[*F*^2^ > 2σ(*F*^2^)], *wR*(*F*^2^), *S*	0.025, 0.066, 1.03
No. of reflections	8619
No. of parameters	385
H-atom treatment	H-atom parameters constrained
Δρ_max_, Δρ_min_ (e Å^−3^)	1.02, −0.50

Computer programs: *CrysAlis PRO* (Rigaku OD, 2024 ▸), *SHELXT* (Sheldrick, 2015*a* ▸), *SHELXL2018/3* (Sheldrick, 2015*b* ▸), *OLEX2* (Dolomanov *et al.*, 2009 ▸) and *publCIF* (Westrip, 2010 ▸).

## full crystallographic data

### Chlorido[(1,2,5,6-η)-cycloocta-1,5-diene](1-ethyl-4-isobutyl-1,2,4-triazol-5-ylidene)rhodium(I) Crystal data

**Table:**

[RhCl(C_8_H_12_)(C_8_H_15_N_3_)]	*Z* = 4
*M**_r_* = 399.76	*F*(000) = 824
Triclinic, *P*1	*D*_x_ = 1.531 Mg m^−^^3^
*a* = 9.6253 (1) Å	Mo *K*α radiation, λ = 0.71073 Å
*b* = 13.6771 (2) Å	Cell parameters from 36515 reflections
*c* = 13.7938 (2) Å	θ = 2.0–28.3°
α = 76.410 (1)°	µ = 1.14 mm^−^^1^
β = 83.455 (1)°	*T* = 100 K
γ = 80.345 (1)°	Block, yellow
*V* = 1734.78 (4) Å^3^	0.3 × 0.23 × 0.15 mm

### Chlorido[(1,2,5,6-η)-cycloocta-1,5-diene](1-ethyl-4-isobutyl-1,2,4-triazol-5-ylidene)rhodium(I) Data collection

**Table:**

Rigaku XtaLAB Synergy-S diffractometer	7885 reflections with *I* > 2σ(*I*)
Detector resolution: 10.0000 pixels mm^-1^	*R*_int_ = 0.037
ω scans	θ_max_ = 28.3°, θ_min_ = 1.9°
Absorption correction: multi-scan (CrysAlisPro; Rigaku OD, 2024)	*h* = −12→12
*T*_min_ = 0.770, *T*_max_ = 1.000	*k* = −18→18
52942 measured reflections	*l* = −17→18
8619 independent reflections	

### Chlorido[(1,2,5,6-η)-cycloocta-1,5-diene](1-ethyl-4-isobutyl-1,2,4-triazol-5-ylidene)rhodium(I) Refinement

**Table:**

Refinement on *F*^2^	0 restraints
Least-squares matrix: full	Hydrogen site location: inferred from neighbouring sites
*R*[*F*^2^ > 2σ(*F*^2^)] = 0.025	H-atom parameters constrained
*wR*(*F*^2^) = 0.066	*w* = 1/[σ^2^(*F*_o_^2^) + (0.0321*P*)^2^ + 2.0009*P*] where *P* = (*F*_o_^2^ + 2*F*_c_^2^)/3
*S* = 1.03	(Δ/σ)_max_ = 0.001
8619 reflections	Δρ_max_ = 1.02 e Å^−^^3^
385 parameters	Δρ_min_ = −0.50 e Å^−^^3^

### Chlorido[(1,2,5,6-η)-cycloocta-1,5-diene](1-ethyl-4-isobutyl-1,2,4-triazol-5-ylidene)rhodium(I) Special details

**Table:**

Geometry. All esds (except the esd in the dihedral angle between two l.s. planes) are estimated using the full covariance matrix. The cell esds are taken into account individually in the estimation of esds in distances, angles and torsion angles; correlations between esds in cell parameters are only used when they are defined by crystal symmetry. An approximate (isotropic) treatment of cell esds is used for estimating esds involving l.s. planes.

### Chlorido[(1,2,5,6-η)-cycloocta-1,5-diene](1-ethyl-4-isobutyl-1,2,4-triazol-5-ylidene)rhodium(I) Fractional atomic coordinates and isotropic or equivalent isotropic displacement parameters (Å^2^)

**Table:**

	*x*	*y*	*z*	*U*_iso_*/*U*_eq_	
Rh1	0.35791 (2)	0.31385 (2)	0.72501 (2)	0.01343 (5)	
Cl1	0.46921 (5)	0.44263 (3)	0.76187 (4)	0.01601 (9)	
N1	0.63831 (18)	0.25377 (13)	0.61135 (13)	0.0179 (3)	
N2	0.76467 (19)	0.18859 (15)	0.61666 (14)	0.0223 (4)	
N3	0.62725 (18)	0.16069 (13)	0.75695 (13)	0.0163 (3)	
C1	0.5521 (2)	0.23940 (15)	0.69543 (15)	0.0154 (4)	
C2	0.7527 (2)	0.13252 (17)	0.70700 (16)	0.0212 (4)	
H2	0.822845	0.078693	0.734610	0.025*	
C3	0.6158 (2)	0.33469 (17)	0.52183 (16)	0.0214 (4)	
H3A	0.514387	0.363675	0.521882	0.026*	
H3B	0.641327	0.305673	0.461753	0.026*	
C4	0.7029 (3)	0.41866 (19)	0.51641 (18)	0.0284 (5)	
H4A	0.681758	0.472773	0.457157	0.043*	
H4B	0.803667	0.391129	0.511948	0.043*	
H4C	0.679746	0.446378	0.576607	0.043*	
C5	0.5869 (2)	0.11712 (16)	0.86246 (15)	0.0184 (4)	
H5A	0.579128	0.044357	0.870410	0.022*	
H5B	0.493144	0.152359	0.882081	0.022*	
C6	0.6947 (2)	0.12717 (16)	0.93138 (15)	0.0183 (4)	
H6	0.789022	0.092337	0.909805	0.022*	
C7	0.7068 (2)	0.23813 (17)	0.92465 (17)	0.0239 (4)	
H7A	0.735865	0.269980	0.855600	0.036*	
H7B	0.777314	0.242137	0.968970	0.036*	
H7C	0.614982	0.273725	0.945043	0.036*	
C8	0.6531 (3)	0.07327 (18)	1.03807 (16)	0.0255 (5)	
H8A	0.559343	0.104992	1.059798	0.038*	
H8B	0.722372	0.078770	1.082634	0.038*	
H8C	0.650800	0.001393	1.040347	0.038*	
C9	0.2598 (2)	0.18390 (16)	0.73460 (17)	0.0211 (4)	
H9	0.326041	0.118340	0.745835	0.025*	
C10	0.2672 (2)	0.24083 (17)	0.63539 (17)	0.0213 (4)	
H10	0.337283	0.207878	0.589157	0.026*	
C11	0.1444 (2)	0.31046 (19)	0.58410 (18)	0.0268 (5)	
H11A	0.081133	0.268428	0.566191	0.032*	
H11B	0.181354	0.353098	0.521253	0.032*	
C12	0.0577 (2)	0.38023 (18)	0.64893 (18)	0.0249 (5)	
H12A	0.012958	0.442801	0.604755	0.030*	
H12B	−0.018645	0.345144	0.689358	0.030*	
C13	0.1475 (2)	0.40917 (16)	0.71818 (17)	0.0200 (4)	
H13	0.150599	0.483800	0.706192	0.024*	
C14	0.1654 (2)	0.35721 (17)	0.81537 (16)	0.0201 (4)	
H14	0.178421	0.401299	0.861133	0.024*	
C15	0.1063 (2)	0.26155 (18)	0.86783 (18)	0.0268 (5)	
H15A	0.004525	0.279333	0.886938	0.032*	
H15B	0.154345	0.231383	0.930027	0.032*	
C16	0.1241 (2)	0.18177 (17)	0.80330 (19)	0.0260 (5)	
H16A	0.125068	0.113287	0.847610	0.031*	
H16B	0.042164	0.194752	0.762303	0.031*	
Rh1'	0.07480 (2)	0.19144 (2)	0.24861 (2)	0.01290 (5)	
Cl1'	0.24607 (5)	0.13510 (4)	0.12636 (3)	0.01603 (9)	
N1'	0.32141 (18)	0.18781 (13)	0.36507 (13)	0.0163 (3)	
N2'	0.42244 (19)	0.24024 (14)	0.38413 (14)	0.0199 (4)	
N3'	0.27156 (18)	0.33698 (13)	0.27928 (13)	0.0161 (3)	
C1'	0.2285 (2)	0.24327 (15)	0.30092 (14)	0.0146 (4)	
C2'	0.3879 (2)	0.33103 (16)	0.33006 (16)	0.0199 (4)	
H2'	0.437503	0.386481	0.326262	0.024*	
C3'	0.3287 (2)	0.07959 (16)	0.40976 (16)	0.0206 (4)	
H3'A	0.241691	0.055764	0.398081	0.025*	
H3'B	0.333658	0.068364	0.482920	0.025*	
C4'	0.4562 (3)	0.01840 (17)	0.36624 (19)	0.0280 (5)	
H4'A	0.449460	0.027066	0.294282	0.042*	
H4'B	0.459312	−0.053659	0.398999	0.042*	
H4'C	0.542437	0.042190	0.377272	0.042*	
C5'	0.2110 (2)	0.42804 (15)	0.20818 (16)	0.0191 (4)	
H5'A	0.251695	0.487350	0.216000	0.023*	
H5'B	0.107738	0.440969	0.224727	0.023*	
C6'	0.2383 (2)	0.41853 (16)	0.09956 (16)	0.0212 (4)	
H6'	0.184237	0.365842	0.089419	0.025*	
C7'	0.3940 (3)	0.3873 (2)	0.07214 (18)	0.0317 (5)	
H7'A	0.449099	0.435015	0.087651	0.048*	
H7'B	0.408573	0.388173	0.000483	0.048*	
H7'C	0.424780	0.318609	0.110580	0.048*	
C8'	0.1823 (3)	0.52123 (18)	0.03371 (19)	0.0342 (6)	
H8'A	0.081581	0.539251	0.052492	0.051*	
H8'B	0.195075	0.516551	−0.036648	0.051*	
H8'C	0.234411	0.573581	0.043154	0.051*	
C9'	−0.0801 (2)	0.28443 (15)	0.32074 (15)	0.0169 (4)	
H9'	−0.041860	0.336934	0.345470	0.020*	
C10'	−0.0575 (2)	0.18603 (15)	0.38251 (15)	0.0174 (4)	
H10'	−0.005804	0.182107	0.442522	0.021*	
C11'	−0.1593 (2)	0.10822 (17)	0.39879 (17)	0.0231 (4)	
H11C	−0.256713	0.144488	0.390277	0.028*	
H11D	−0.156544	0.067389	0.468255	0.028*	
C12'	−0.1232 (2)	0.03646 (17)	0.32576 (18)	0.0243 (5)	
H12C	−0.055371	−0.022884	0.355491	0.029*	
H12D	−0.210286	0.011085	0.316728	0.029*	
C13'	−0.0602 (2)	0.08679 (16)	0.22444 (16)	0.0196 (4)	
H13'	−0.012685	0.038113	0.182806	0.023*	
C14'	−0.1080 (2)	0.18225 (17)	0.16998 (16)	0.0205 (4)	
H14'	−0.088014	0.190447	0.095986	0.025*	
C15'	−0.2389 (2)	0.25045 (18)	0.20069 (18)	0.0241 (5)	
H15C	−0.304485	0.208130	0.245477	0.029*	
H15D	−0.287981	0.287450	0.140498	0.029*	
C16'	−0.2006 (2)	0.32711 (16)	0.25459 (16)	0.0216 (4)	
H16C	−0.174614	0.386987	0.204044	0.026*	
H16D	−0.284902	0.350564	0.295974	0.026*	

### Chlorido[(1,2,5,6-η)-cycloocta-1,5-diene](1-ethyl-4-isobutyl-1,2,4-triazol-5-ylidene)rhodium(I) Atomic displacement parameters (Å^2^)

**Table:**

	*U*^11^	*U*^22^	*U*^33^	*U*^12^	*U*^13^	*U*^23^
Rh1	0.01197 (8)	0.01211 (8)	0.01682 (8)	−0.00219 (5)	−0.00155 (5)	−0.00392 (5)
Cl1	0.0141 (2)	0.00959 (19)	0.0263 (2)	−0.00364 (16)	−0.00341 (17)	−0.00568 (17)
N1	0.0145 (8)	0.0195 (8)	0.0194 (8)	−0.0007 (7)	−0.0017 (6)	−0.0050 (7)
N2	0.0173 (9)	0.0253 (9)	0.0232 (9)	0.0030 (7)	−0.0012 (7)	−0.0080 (7)
N3	0.0149 (8)	0.0149 (8)	0.0191 (8)	−0.0008 (6)	−0.0019 (6)	−0.0047 (6)
C1	0.0161 (9)	0.0136 (9)	0.0184 (9)	−0.0043 (7)	−0.0020 (7)	−0.0056 (7)
C2	0.0172 (10)	0.0220 (10)	0.0247 (11)	0.0017 (8)	−0.0021 (8)	−0.0088 (8)
C3	0.0209 (10)	0.0252 (11)	0.0169 (10)	−0.0033 (8)	−0.0027 (8)	−0.0016 (8)
C4	0.0312 (12)	0.0287 (12)	0.0240 (11)	−0.0109 (10)	−0.0004 (9)	0.0003 (9)
C5	0.0187 (10)	0.0164 (9)	0.0191 (10)	−0.0048 (8)	−0.0014 (8)	−0.0007 (7)
C6	0.0163 (9)	0.0174 (9)	0.0206 (10)	−0.0024 (7)	−0.0025 (8)	−0.0028 (8)
C7	0.0254 (11)	0.0229 (11)	0.0253 (11)	−0.0077 (9)	−0.0052 (9)	−0.0047 (9)
C8	0.0266 (11)	0.0284 (12)	0.0209 (10)	−0.0092 (9)	−0.0040 (9)	0.0007 (9)
C9	0.0184 (10)	0.0169 (10)	0.0313 (11)	−0.0063 (8)	−0.0030 (8)	−0.0087 (8)
C10	0.0181 (10)	0.0224 (10)	0.0278 (11)	−0.0034 (8)	−0.0040 (8)	−0.0130 (9)
C11	0.0239 (11)	0.0333 (12)	0.0267 (11)	−0.0037 (9)	−0.0092 (9)	−0.0104 (10)
C12	0.0187 (10)	0.0267 (11)	0.0290 (12)	−0.0002 (9)	−0.0076 (9)	−0.0048 (9)
C13	0.0128 (9)	0.0180 (10)	0.0289 (11)	0.0019 (7)	−0.0010 (8)	−0.0076 (8)
C14	0.0157 (9)	0.0223 (10)	0.0229 (10)	−0.0012 (8)	0.0017 (8)	−0.0088 (8)
C15	0.0214 (11)	0.0294 (12)	0.0272 (11)	−0.0070 (9)	0.0043 (9)	−0.0023 (9)
C16	0.0199 (10)	0.0213 (11)	0.0359 (12)	−0.0078 (8)	−0.0003 (9)	−0.0021 (9)
Rh1'	0.01226 (8)	0.01255 (8)	0.01436 (8)	−0.00244 (5)	−0.00160 (5)	−0.00315 (5)
Cl1'	0.0145 (2)	0.0157 (2)	0.0178 (2)	−0.00018 (16)	0.00186 (17)	−0.00625 (17)
N1'	0.0162 (8)	0.0159 (8)	0.0177 (8)	−0.0057 (6)	−0.0035 (6)	−0.0020 (6)
N2'	0.0183 (8)	0.0200 (9)	0.0233 (9)	−0.0077 (7)	−0.0054 (7)	−0.0035 (7)
N3'	0.0165 (8)	0.0149 (8)	0.0170 (8)	−0.0050 (6)	−0.0014 (6)	−0.0021 (6)
C1'	0.0154 (9)	0.0139 (9)	0.0143 (9)	−0.0025 (7)	0.0012 (7)	−0.0039 (7)
C2'	0.0186 (10)	0.0203 (10)	0.0225 (10)	−0.0075 (8)	−0.0027 (8)	−0.0039 (8)
C3'	0.0223 (10)	0.0156 (10)	0.0228 (10)	−0.0061 (8)	−0.0075 (8)	0.0029 (8)
C4'	0.0267 (12)	0.0189 (11)	0.0391 (13)	−0.0010 (9)	−0.0125 (10)	−0.0048 (9)
C5'	0.0201 (10)	0.0132 (9)	0.0229 (10)	−0.0031 (8)	−0.0018 (8)	−0.0013 (8)
C6'	0.0290 (11)	0.0139 (9)	0.0209 (10)	−0.0038 (8)	−0.0056 (8)	−0.0020 (8)
C7'	0.0355 (13)	0.0318 (13)	0.0243 (12)	−0.0035 (10)	0.0067 (10)	−0.0046 (10)
C8'	0.0565 (17)	0.0180 (11)	0.0267 (12)	−0.0020 (11)	−0.0129 (11)	0.0002 (9)
C9'	0.0163 (9)	0.0172 (9)	0.0183 (9)	−0.0035 (7)	0.0022 (7)	−0.0073 (7)
C10'	0.0180 (9)	0.0182 (10)	0.0168 (9)	−0.0057 (8)	0.0018 (7)	−0.0049 (7)
C11'	0.0234 (11)	0.0214 (10)	0.0260 (11)	−0.0105 (9)	0.0043 (9)	−0.0062 (8)
C12'	0.0209 (10)	0.0198 (10)	0.0343 (12)	−0.0083 (8)	0.0044 (9)	−0.0092 (9)
C13'	0.0163 (9)	0.0211 (10)	0.0262 (11)	−0.0052 (8)	−0.0025 (8)	−0.0127 (8)
C14'	0.0165 (10)	0.0259 (11)	0.0227 (10)	−0.0025 (8)	−0.0054 (8)	−0.0111 (8)
C15'	0.0163 (10)	0.0277 (11)	0.0295 (12)	0.0024 (8)	−0.0072 (9)	−0.0098 (9)
C16'	0.0198 (10)	0.0196 (10)	0.0239 (11)	0.0019 (8)	−0.0004 (8)	−0.0059 (8)

### Chlorido[(1,2,5,6-η)-cycloocta-1,5-diene](1-ethyl-4-isobutyl-1,2,4-triazol-5-ylidene)rhodium(I) Geometric parameters (Å, º)

**Table:**

Rh1—Cl1	2.3846 (5)	Rh1'—Cl1'	2.3887 (5)
Rh1—C1	2.020 (2)	Rh1'—C1'	2.012 (2)
Rh1—C9	2.120 (2)	Rh1'—C9'	2.110 (2)
Rh1—C10	2.099 (2)	Rh1'—C10'	2.114 (2)
Rh1—C13	2.216 (2)	Rh1'—C13'	2.190 (2)
Rh1—C14	2.189 (2)	Rh1'—C14'	2.205 (2)
N1—N2	1.380 (2)	N1'—N2'	1.382 (2)
N1—C1	1.342 (3)	N1'—C1'	1.340 (3)
N1—C3	1.461 (3)	N1'—C3'	1.457 (3)
N2—C2	1.305 (3)	N2'—C2'	1.301 (3)
N3—C1	1.361 (3)	N3'—C1'	1.369 (2)
N3—C2	1.364 (3)	N3'—C2'	1.369 (3)
N3—C5	1.471 (3)	N3'—C5'	1.471 (3)
C2—H2	0.9500	C2'—H2'	0.9500
C3—H3A	0.9900	C3'—H3'A	0.9900
C3—H3B	0.9900	C3'—H3'B	0.9900
C3—C4	1.515 (3)	C3'—C4'	1.514 (3)
C4—H4A	0.9800	C4'—H4'A	0.9800
C4—H4B	0.9800	C4'—H4'B	0.9800
C4—H4C	0.9800	C4'—H4'C	0.9800
C5—H5A	0.9900	C5'—H5'A	0.9900
C5—H5B	0.9900	C5'—H5'B	0.9900
C5—C6	1.527 (3)	C5'—C6'	1.524 (3)
C6—H6	1.0000	C6'—H6'	1.0000
C6—C7	1.522 (3)	C6'—C7'	1.517 (3)
C6—C8	1.525 (3)	C6'—C8'	1.532 (3)
C7—H7A	0.9800	C7'—H7'A	0.9800
C7—H7B	0.9800	C7'—H7'B	0.9800
C7—H7C	0.9800	C7'—H7'C	0.9800
C8—H8A	0.9800	C8'—H8'A	0.9800
C8—H8B	0.9800	C8'—H8'B	0.9800
C8—H8C	0.9800	C8'—H8'C	0.9800
C9—H9	1.0000	C9'—H9'	1.0000
C9—C10	1.407 (3)	C9'—C10'	1.411 (3)
C9—C16	1.524 (3)	C9'—C16'	1.511 (3)
C10—H10	1.0000	C10'—H10'	1.0000
C10—C11	1.517 (3)	C10'—C11'	1.527 (3)
C11—H11A	0.9900	C11'—H11C	0.9900
C11—H11B	0.9900	C11'—H11D	0.9900
C11—C12	1.540 (3)	C11'—C12'	1.539 (3)
C12—H12A	0.9900	C12'—H12C	0.9900
C12—H12B	0.9900	C12'—H12D	0.9900
C12—C13	1.516 (3)	C12'—C13'	1.513 (3)
C13—H13	1.0000	C13'—H13'	1.0000
C13—C14	1.377 (3)	C13'—C14'	1.377 (3)
C14—H14	1.0000	C14'—H14'	1.0000
C14—C15	1.507 (3)	C14'—C15'	1.519 (3)
C15—H15A	0.9900	C15'—H15C	0.9900
C15—H15B	0.9900	C15'—H15D	0.9900
C15—C16	1.540 (3)	C15'—C16'	1.532 (3)
C16—H16A	0.9900	C16'—H16C	0.9900
C16—H16B	0.9900	C16'—H16D	0.9900
			
C1—Rh1—Cl1	88.36 (5)	C1'—Rh1'—Cl1'	88.57 (6)
C1—Rh1—C9	92.59 (8)	C1'—Rh1'—C9'	90.29 (8)
C1—Rh1—C10	91.43 (8)	C1'—Rh1'—C10'	93.67 (8)
C1—Rh1—C13	166.29 (8)	C1'—Rh1'—C13'	160.34 (8)
C1—Rh1—C14	157.23 (8)	C1'—Rh1'—C14'	163.12 (8)
C9—Rh1—Cl1	164.45 (6)	C9'—Rh1'—Cl1'	161.60 (6)
C9—Rh1—C13	89.51 (8)	C9'—Rh1'—C10'	39.02 (8)
C9—Rh1—C14	81.88 (8)	C9'—Rh1'—C13'	98.19 (8)
C10—Rh1—Cl1	156.57 (6)	C9'—Rh1'—C14'	81.70 (8)
C10—Rh1—C9	38.96 (9)	C10'—Rh1'—Cl1'	159.36 (6)
C10—Rh1—C13	81.72 (8)	C10'—Rh1'—C13'	82.22 (8)
C10—Rh1—C14	97.75 (8)	C10'—Rh1'—C14'	89.42 (8)
C13—Rh1—Cl1	93.23 (6)	C13'—Rh1'—Cl1'	88.80 (6)
C14—Rh1—Cl1	91.27 (6)	C13'—Rh1'—C14'	36.52 (8)
C14—Rh1—C13	36.43 (8)	C14'—Rh1'—Cl1'	94.37 (6)
N2—N1—C3	119.00 (17)	N2'—N1'—C3'	119.18 (16)
C1—N1—N2	114.16 (17)	C1'—N1'—N2'	114.54 (16)
C1—N1—C3	126.62 (18)	C1'—N1'—C3'	126.18 (17)
C2—N2—N1	102.62 (17)	C2'—N2'—N1'	102.61 (16)
C1—N3—C2	108.75 (17)	C1'—N3'—C5'	126.56 (17)
C1—N3—C5	126.42 (17)	C2'—N3'—C1'	108.60 (17)
C2—N3—C5	124.68 (18)	C2'—N3'—C5'	124.71 (17)
N1—C1—Rh1	129.32 (15)	N1'—C1'—Rh1'	125.97 (14)
N1—C1—N3	102.77 (17)	N1'—C1'—N3'	102.45 (16)
N3—C1—Rh1	127.91 (14)	N3'—C1'—Rh1'	131.50 (15)
N2—C2—N3	111.69 (19)	N2'—C2'—N3'	111.78 (18)
N2—C2—H2	124.2	N2'—C2'—H2'	124.1
N3—C2—H2	124.2	N3'—C2'—H2'	124.1
N1—C3—H3A	109.2	N1'—C3'—H3'A	109.3
N1—C3—H3B	109.2	N1'—C3'—H3'B	109.3
N1—C3—C4	111.92 (18)	N1'—C3'—C4'	111.53 (18)
H3A—C3—H3B	107.9	H3'A—C3'—H3'B	108.0
C4—C3—H3A	109.2	C4'—C3'—H3'A	109.3
C4—C3—H3B	109.2	C4'—C3'—H3'B	109.3
C3—C4—H4A	109.5	C3'—C4'—H4'A	109.5
C3—C4—H4B	109.5	C3'—C4'—H4'B	109.5
C3—C4—H4C	109.5	C3'—C4'—H4'C	109.5
H4A—C4—H4B	109.5	H4'A—C4'—H4'B	109.5
H4A—C4—H4C	109.5	H4'A—C4'—H4'C	109.5
H4B—C4—H4C	109.5	H4'B—C4'—H4'C	109.5
N3—C5—H5A	109.3	N3'—C5'—H5'A	108.9
N3—C5—H5B	109.3	N3'—C5'—H5'B	108.9
N3—C5—C6	111.69 (16)	N3'—C5'—C6'	113.21 (17)
H5A—C5—H5B	107.9	H5'A—C5'—H5'B	107.7
C6—C5—H5A	109.3	C6'—C5'—H5'A	108.9
C6—C5—H5B	109.3	C6'—C5'—H5'B	108.9
C5—C6—H6	108.3	C5'—C6'—H6'	108.7
C7—C6—C5	111.33 (17)	C5'—C6'—C8'	107.67 (18)
C7—C6—H6	108.3	C7'—C6'—C5'	111.82 (19)
C7—C6—C8	111.59 (18)	C7'—C6'—H6'	108.7
C8—C6—C5	109.01 (17)	C7'—C6'—C8'	111.1 (2)
C8—C6—H6	108.3	C8'—C6'—H6'	108.7
C6—C7—H7A	109.5	C6'—C7'—H7'A	109.5
C6—C7—H7B	109.5	C6'—C7'—H7'B	109.5
C6—C7—H7C	109.5	C6'—C7'—H7'C	109.5
H7A—C7—H7B	109.5	H7'A—C7'—H7'B	109.5
H7A—C7—H7C	109.5	H7'A—C7'—H7'C	109.5
H7B—C7—H7C	109.5	H7'B—C7'—H7'C	109.5
C6—C8—H8A	109.5	C6'—C8'—H8'A	109.5
C6—C8—H8B	109.5	C6'—C8'—H8'B	109.5
C6—C8—H8C	109.5	C6'—C8'—H8'C	109.5
H8A—C8—H8B	109.5	H8'A—C8'—H8'B	109.5
H8A—C8—H8C	109.5	H8'A—C8'—H8'C	109.5
H8B—C8—H8C	109.5	H8'B—C8'—H8'C	109.5
Rh1—C9—H9	114.1	Rh1'—C9'—H9'	113.9
C10—C9—Rh1	69.75 (12)	C10'—C9'—Rh1'	70.67 (12)
C10—C9—H9	114.1	C10'—C9'—H9'	113.9
C10—C9—C16	123.7 (2)	C10'—C9'—C16'	126.31 (19)
C16—C9—Rh1	113.47 (15)	C16'—C9'—Rh1'	109.39 (14)
C16—C9—H9	114.1	C16'—C9'—H9'	113.9
Rh1—C10—H10	113.9	Rh1'—C10'—H10'	113.7
C9—C10—Rh1	71.29 (12)	C9'—C10'—Rh1'	70.32 (11)
C9—C10—H10	113.9	C9'—C10'—H10'	113.7
C9—C10—C11	125.0 (2)	C9'—C10'—C11'	124.48 (19)
C11—C10—Rh1	110.99 (15)	C11'—C10'—Rh1'	113.29 (14)
C11—C10—H10	113.9	C11'—C10'—H10'	113.7
C10—C11—H11A	108.9	C10'—C11'—H11C	109.1
C10—C11—H11B	108.9	C10'—C11'—H11D	109.1
C10—C11—C12	113.42 (18)	C10'—C11'—C12'	112.48 (17)
H11A—C11—H11B	107.7	H11C—C11'—H11D	107.8
C12—C11—H11A	108.9	C12'—C11'—H11C	109.1
C12—C11—H11B	108.9	C12'—C11'—H11D	109.1
C11—C12—H12A	109.1	C11'—C12'—H12C	108.9
C11—C12—H12B	109.1	C11'—C12'—H12D	108.9
H12A—C12—H12B	107.9	H12C—C12'—H12D	107.7
C13—C12—C11	112.38 (18)	C13'—C12'—C11'	113.21 (18)
C13—C12—H12A	109.1	C13'—C12'—H12C	108.9
C13—C12—H12B	109.1	C13'—C12'—H12D	108.9
Rh1—C13—H13	114.2	Rh1'—C13'—H13'	114.2
C12—C13—Rh1	111.23 (14)	C12'—C13'—Rh1'	107.61 (14)
C12—C13—H13	114.2	C12'—C13'—H13'	114.2
C14—C13—Rh1	70.71 (12)	C14'—C13'—Rh1'	72.31 (12)
C14—C13—C12	124.2 (2)	C14'—C13'—C12'	125.9 (2)
C14—C13—H13	114.2	C14'—C13'—H13'	114.2
Rh1—C14—H14	113.9	Rh1'—C14'—H14'	114.1
C13—C14—Rh1	72.87 (12)	C13'—C14'—Rh1'	71.16 (12)
C13—C14—H14	113.9	C13'—C14'—H14'	114.1
C13—C14—C15	126.2 (2)	C13'—C14'—C15'	124.4 (2)
C15—C14—Rh1	107.91 (14)	C15'—C14'—Rh1'	111.23 (14)
C15—C14—H14	113.9	C15'—C14'—H14'	114.1
C14—C15—H15A	108.9	C14'—C15'—H15C	109.4
C14—C15—H15B	108.9	C14'—C15'—H15D	109.4
C14—C15—C16	113.18 (19)	C14'—C15'—C16'	111.22 (17)
H15A—C15—H15B	107.8	H15C—C15'—H15D	108.0
C16—C15—H15A	108.9	C16'—C15'—H15C	109.4
C16—C15—H15B	108.9	C16'—C15'—H15D	109.4
C9—C16—C15	112.07 (18)	C9'—C16'—C15'	113.46 (18)
C9—C16—H16A	109.2	C9'—C16'—H16C	108.9
C9—C16—H16B	109.2	C9'—C16'—H16D	108.9
C15—C16—H16A	109.2	C15'—C16'—H16C	108.9
C15—C16—H16B	109.2	C15'—C16'—H16D	108.9
H16A—C16—H16B	107.9	H16C—C16'—H16D	107.7
			
Rh1—C9—C10—C11	−103.0 (2)	Rh1'—C9'—C10'—C11'	−105.3 (2)
Rh1—C9—C16—C15	−12.6 (2)	Rh1'—C9'—C16'—C15'	39.8 (2)
Rh1—C10—C11—C12	−35.7 (2)	Rh1'—C10'—C11'—C12'	11.5 (2)
Rh1—C13—C14—C15	−100.0 (2)	Rh1'—C13'—C14'—C15'	−103.3 (2)
Rh1—C14—C15—C16	−37.0 (2)	Rh1'—C14'—C15'—C16'	15.3 (2)
N1—N2—C2—N3	−0.6 (2)	N1'—N2'—C2'—N3'	0.1 (2)
N2—N1—C1—Rh1	−179.85 (14)	N2'—N1'—C1'—Rh1'	175.92 (14)
N2—N1—C1—N3	−0.4 (2)	N2'—N1'—C1'—N3'	−1.1 (2)
N2—N1—C3—C4	−72.5 (2)	N2'—N1'—C3'—C4'	−67.9 (2)
N3—C5—C6—C7	61.0 (2)	N3'—C5'—C6'—C7'	−52.5 (2)
N3—C5—C6—C8	−175.51 (17)	N3'—C5'—C6'—C8'	−174.82 (19)
C1—N1—N2—C2	0.6 (2)	C1'—N1'—N2'—C2'	0.6 (2)
C1—N1—C3—C4	101.8 (2)	C1'—N1'—C3'—C4'	108.2 (2)
C1—N3—C2—N2	0.5 (3)	C1'—N3'—C2'—N2'	−0.8 (3)
C1—N3—C5—C6	−118.8 (2)	C1'—N3'—C5'—C6'	−68.1 (3)
C2—N3—C1—Rh1	179.44 (14)	C2'—N3'—C1'—Rh1'	−175.68 (16)
C2—N3—C1—N1	−0.1 (2)	C2'—N3'—C1'—N1'	1.1 (2)
C2—N3—C5—C6	56.2 (3)	C2'—N3'—C5'—C6'	107.3 (2)
C3—N1—N2—C2	175.61 (19)	C3'—N1'—N2'—C2'	177.21 (19)
C3—N1—C1—Rh1	5.6 (3)	C3'—N1'—C1'—Rh1'	−0.4 (3)
C3—N1—C1—N3	−174.88 (19)	C3'—N1'—C1'—N3'	−177.37 (19)
C5—N3—C1—Rh1	−4.9 (3)	C5'—N3'—C1'—Rh1'	0.3 (3)
C5—N3—C1—N1	175.64 (18)	C5'—N3'—C1'—N1'	177.04 (18)
C5—N3—C2—N2	−175.31 (18)	C5'—N3'—C2'—N2'	−176.84 (18)
C9—C10—C11—C12	45.6 (3)	C9'—C10'—C11'—C12'	92.9 (3)
C10—C9—C16—C15	−93.2 (3)	C10'—C9'—C16'—C15'	−40.0 (3)
C10—C11—C12—C13	31.7 (3)	C10'—C11'—C12'—C13'	−32.6 (3)
C11—C12—C13—Rh1	−12.7 (2)	C11'—C12'—C13'—Rh1'	36.5 (2)
C11—C12—C13—C14	−93.2 (3)	C11'—C12'—C13'—C14'	−44.0 (3)
C12—C13—C14—Rh1	103.1 (2)	C12'—C13'—C14'—Rh1'	99.3 (2)
C12—C13—C14—C15	3.1 (3)	C12'—C13'—C14'—C15'	−4.0 (3)
C13—C14—C15—C16	44.6 (3)	C13'—C14'—C15'—C16'	96.4 (2)
C14—C15—C16—C9	33.5 (3)	C14'—C15'—C16'—C9'	−36.4 (3)
C16—C9—C10—Rh1	105.3 (2)	C16'—C9'—C10'—Rh1'	100.3 (2)
C16—C9—C10—C11	2.4 (3)	C16'—C9'—C10'—C11'	−5.0 (3)

### Chlorido[(1,2,5,6-η)-cycloocta-1,5-diene](1-ethyl-4-isobutyl-1,2,4-triazol-5-ylidene)rhodium(I) Hydrogen-bond geometry (Å, º)

**Table:**

*D*—H···*A*	*D*—H	H···*A*	*D*···*A*	*D*—H···*A*
C2′—H2′···Cl1^i^	0.95	2.62	3.502 (2)	155

Symmetry code: (i) −*x*+1, −*y*+1, −*z*+1.
